# Antibody Conjugates for Targeted Therapy Against HIV-1 as an Emerging Tool for HIV-1 Cure

**DOI:** 10.3389/fimmu.2021.708806

**Published:** 2021-07-01

**Authors:** Jeffrey C. Umotoy, Steven W. de Taeye

**Affiliations:** Laboratory of Experimental Virology, Department of Medical Microbiology, Amsterdam University Medical Center (UMC), Amsterdam Infection and Immunity Institute, University of Amsterdam, Amsterdam, Netherlands

**Keywords:** antibody conjugates, immunoconjugates, HIV-1 cure, ADC, HIV-1 antibody conjugates, HIV-1 ADC

## Abstract

Although advances in antiretroviral therapy (ART) have significantly improved the life expectancy of people living with HIV-1 (PLWH) by suppressing HIV-1 replication, a cure for HIV/AIDS remains elusive. Recent findings of the emergence of drug resistance against various ART have resulted in an increased number of treatment failures, thus the development of novel strategies for HIV-1 cure is of immediate need. Antibody-based therapy is a well-established tool in the treatment of various diseases and the engineering of new antibody derivatives is expanding the realms of its application. An antibody-based carrier of anti-HIV-1 molecules, or antibody conjugates (ACs), could address the limitations of current HIV-1 ART by decreasing possible off-target effects, reduce toxicity, increasing the therapeutic index, and lowering production costs. Broadly neutralizing antibodies (bNAbs) with exceptional breadth and potency against HIV-1 are currently being explored to prevent or treat HIV-1 infection in the clinic. Moreover, bNAbs can be engineered to deliver cytotoxic or immune regulating molecules as ACs, further increasing its therapeutic potential for HIV-1 cure. ACs are currently an important component of anticancer treatment with several FDA-approved constructs, however, to date, no ACs are approved to treat viral infections. This review aims to outline the development of AC for HIV-1 cure, examine the variety of carriers and payloads used, and discuss the potential of ACs in the current HIV-1 cure landscape.

## Introduction

Decades after the discovery of HIV-1 as the causative agent for AIDS, no vaccine or curative treatment is available against HIV-1 infection. Although the advent of antiretroviral therapy (ART) has significantly improved the disease outcome of people living with HIV-1 (PLWH) from a disease with high morbidity and mortality to a manageable chronic disease, HIV-1 remains incurable. In 2019, an estimated 67% of HIV-1 infected individuals were undergoing ART out of the estimated 38 million people infected with HIV-1 globally. The effectiveness of ART regimen is unequivocal in keeping viral loads to undetectable levels, reduction of transmissions, and the overall impact on the life expectancy of PLWH, however, life-long ART is linked to new sets of challenges. Accessibility remains an issue with an estimated 12 million people without access to ART in 2019, as well as the issue of adherence to daily drug intake for people undergoing treatment (UNAIDS data 2020). More alarming is the increasing evidence of drug resistance in the current ART, which could undermine the advances achieved thus far. Therefore, an innovative therapeutic approach for a functional or total cure is needed to fully end the AIDS epidemic. One such focus is the engineering of a highly targeted delivery system of molecular payload for HIV-1 to target host cells acutely or latently infected with HIV.

**Figure 1:**
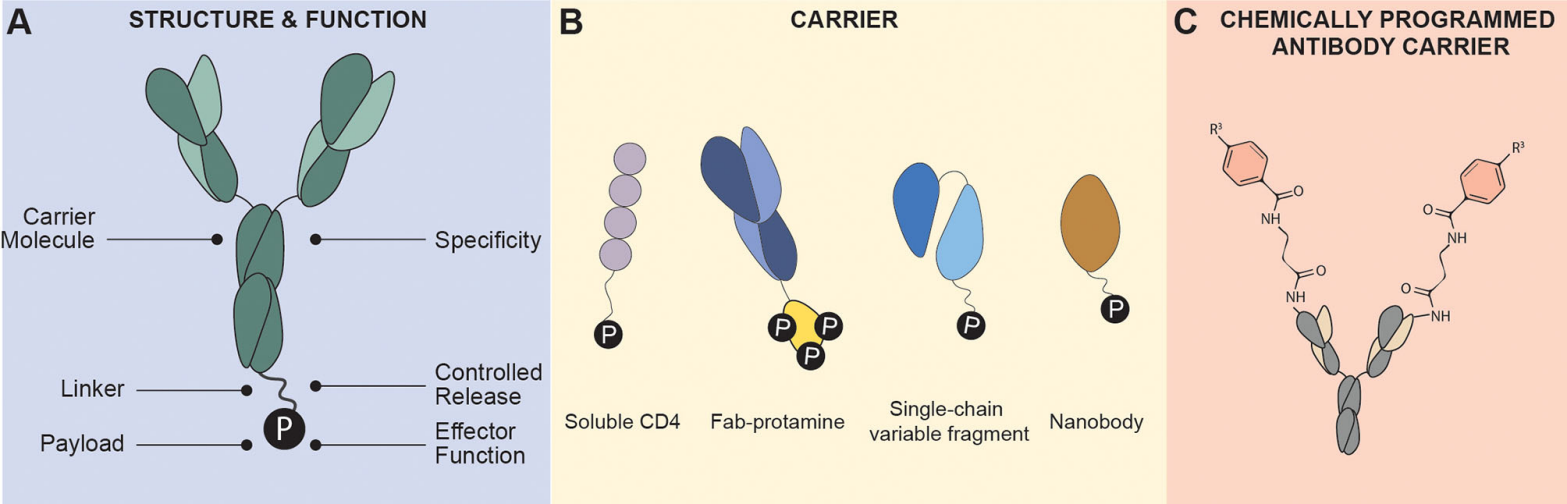
Antibody Conjugate Structure. **(A)** Antibody conjugates are characterized by a fusion of a carrier molecule specific to an antigen for specificity, a linker that connects the carrier and payload providing flexibility and controlled release of payload, and the effector molecule “payload” that can induce cytotoxicity or immune modulation. **(B)** Carrier molecules are typically monoclonal antibodies (such as IgG1), soluble ligands such as cytokines or receptors), Fab fragment, single-chain variable fragment, and nanobodies. **(C)** Chemically programmed antibody containing a reactive residue on its binding pocket that can react to derivatives of small molecules. Targeting is conferred by the conjugated small molecules instead of the antibody.

Antibody conjugates (ACs) are engineered therapeutics designed with a targeting “carrier” moiety, such as monoclonal antibodies (mAb), conjugated to a cytotoxic or immune regulating molecular “payload” connected *via* a linker (**Figure 1**). In cancer immunotherapy, the targeting molecule is directed against a specific disease-related antigen expressed on cells to which the cytotoxic drug payload is delivered inducing cell death. The main goal of targeted drug delivery is to optimize a payload’s therapeutic index by localizing its pharmacological activity only to sites expressing the antigen target. The conjugation of the carrier and payload subunits are mutually beneficial: improved cytocidal effect for the antibody carrier, while increased systemic half-life, and reduced off-target toxicities for the payload. The cancer immunotherapy field is dominating the AC landscape with 12 FDA-approved ACs used for the treatment of various cancers (antibodysociety.org).

To date, no ACs for antiviral use are approved for therapy. Although advances and challenges in the design of AC for cancer immunotherapy can, in theory, be applied to ACs for antiviral use, a different set of challenges must be overcome for HIV-1. Some challenges in ACs for HIV-1 that are unique from cancers are (a) high variability of viral antigen expressed on infected cells, (b) release of cell-free virions, (c) persistence of infected cells in the blood and lymphoid tissues, (d) infection of anatomic “sanctuary sites”, and (f) the persistence of latently infected cells which are void of any viral surface protein expression until after reactivation.

This review will focus on the diverse AC constructs designed for HIV-1 cure including the variety of carrier molecules, effector payloads, and the choice of viral or host antigen targets (**Table 1**). This review will not focus on the broader AC landscape such as conjugation chemistries, linker design, and biodistribution, which has been reviewed extensively elsewhere (1–5). Instead, this review will provide insights into the development of the next-generation ACs for HIV-1 cure based on the use of novel antibody carriers such as broadly neutralizing antibodies (bNAbs) and their subunit derivatives (**Figure 1B**), combination ART and AC treatment, and how the AC landscape can advance to human clinical trials for HIV-1 cure.

**Table 1:** Antibody conjugate categories.

Antibody Conjugate	Payload	Description
Antibody-toxin conjugates	Toxin	Commonly referred to as immunotoxin in the cancer immunotherapy field, ATC is characterized by the conjugation of toxins usually derived from plants or bacteria.
Antibody-radionuclide conjugates	Radionuclide	Antibody-radionuclide conjugate is the conjugation of a radionuclide to an antibody carrier
Antibody-drug conjugates	Small molecule or compounds	Antibody-drug conjugate is the conjugation of small molecule drugs to antibodies.
Antibody-oligonucleotide conjugates	Oligonucleotides such as siRNA	Conjugation of oligonucleotide-based payloads such as siRNA
Antibody-photosensitizer	Photosensitizers	Antibody-photosensitizer conjugates, also known as photoimmunotherapy (PIT), is a targeted photodynamic therapy designed with a conjugated photosensitizer to a mAb.

## HIV Latency

HIV-1 persists in all subsets of memory CD4+ T cells as well as a subset of functional T cells, such as T follicular helper cells, T regulatory cells, Th1 cells, and Th17 cells (6). Besides these cellular reservoirs, HIV-1 can persist latently in tissue reservoirs throughout the body (7, 8). Latency is characterized by the presence of integrated but transcriptionally silent HIV-1 DNA, undetectability from immune surveillance, and resistance to ART (9–12). Current ART interferes at various stages of the HIV-1 replication cycle and has been shown to effectively control viral loads to undetectable levels (13). However, ART fails to eliminate or reduce the viral reservoir of latently infected cells, and upon ART cessation, a rapid viral rebound is observed (14). Therefore, major efforts in the HIV-1 cure field aims at eliminating the viral reservoir of latently infected cells. The two main curative approaches for HIV-1 cure include the shock-and-kill and block-and-lock approach.

The shock-and-kill approach aims to reactivate or “shock” the transcriptionally silent host-integrated HIV-1 DNA in latently infected cells *via* latency-reversing agents (LRAs). Upon reactivation, the “kill” stage occurs either through host cytopathic effects or *via* induction of immune-mediated clearance (15). Concurrent ART simultaneously addresses the suppression of infection. A variety of LRAs has been described to reactivate latently infected cells. The most studied LRAs either activate the NF-κb signaling pathway or inhibit the epigenetic writers, but a plethora of LRAs belonging to various functional categories are growing (16). The shock-and-kill approach, therefore, relies heavily on the induction of *de novo* HIV-1 protein expression upon latency reversal with LRAs, permitting immune recognition and killing. In initial clinical trials, an increase in cellular HIV-1 RNA after LRA treatment was observed indicating latency reversal, however, no decrease in the overall reservoir size was observed (17, 18). The failure to reduce the reservoir size, therefore, indicates that latency reversal must be combined with therapies that can augment antiviral immune responses. To boost the “killing” phase of shock-and-kill, various therapeutic approaches are currently under consideration, such as immunomodulatory therapies, CAR-T/NK therapy, therapeutic HIV-1 vaccinations, and various antibody-based strategies (19, 20).

The failure to eradicate the viral reservoir size through the shock-and-kill approach encouraged the re-evaluation of the definition of HIV cure. The total cure strategy (also referred to as sterilizing cure), centered around the eradication of all latently infected cells, proved to be challenging and a more feasible “functional cure” is proposed, which is characterized by a long-term HIV-1 remission (21). One such approach is the block-and-lock strategy that aims to prevent viral reactivation in latently infected cells for a prolonged drug-free remission (22, 23). This approach targets either HIV-1 or host-specific factors to induce a state of deep or permanent latency in the absence of ART. As a counterpart to LRAs, the block-and-lock approach uses latency-promoting agents (LPAs) to promote “blocking” of virus transcription and “locking” the virus promoter in a deep latent state *via* repressive epigenetic modifications (24–26).

The quest to finding an HIV-1 cure, either a total or functional cure, continues. It is likely that monotherapy will not be sufficient to address the complexities of HIV-1 infection, and a multi-pronged approach involving combination ART, LRAs/LPAs, and immunotherapies are needed.

## Target of HIV-1 Specific Antibody Conjugates

**Figure 2:**
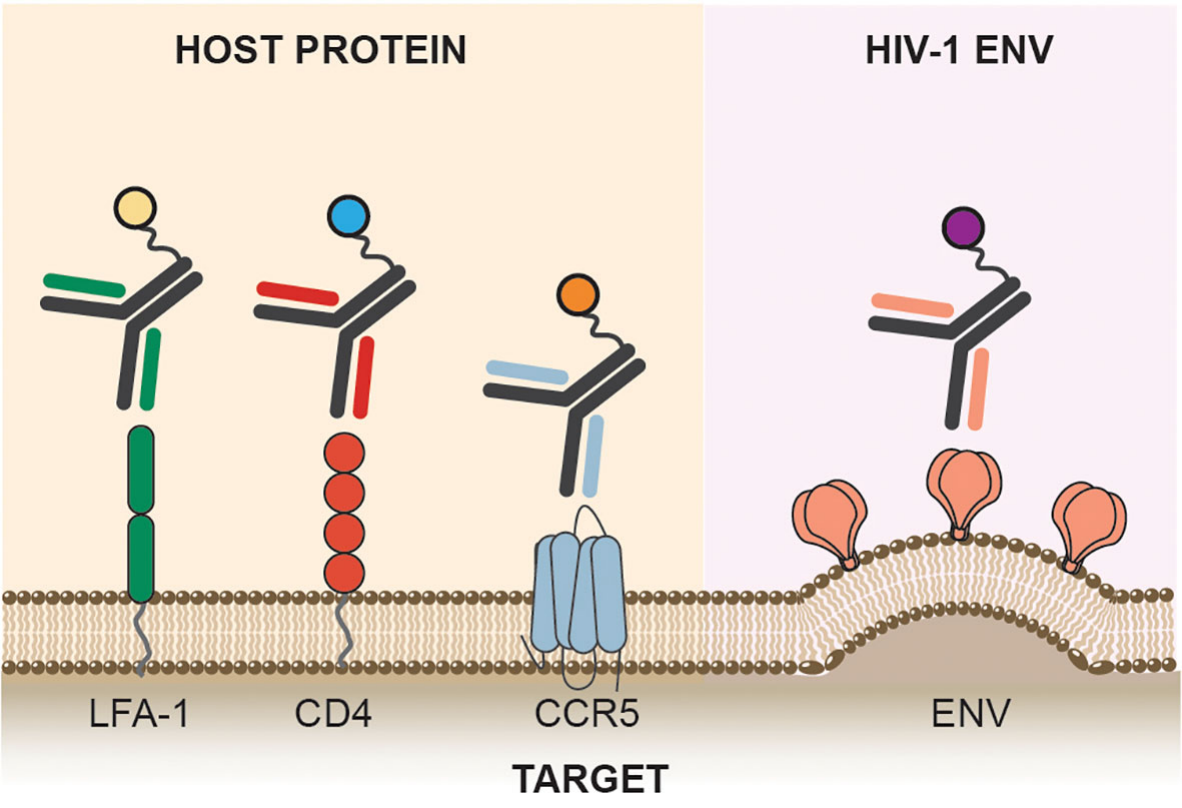
Targets. Antibody conjugates can target host receptors required for viral entry, CD4, and coreceptor such as CCR5. Host factors such as LFA-1, an integrin protein expressed on lymphocytes are also targeted by antibody conjugates. HIV-1 Env, the sole viral protein expressed on infected cells is also the targets of antibody conjugates.

To deliver cytotoxic or immune-modulating molecules to HIV-1 infected cells, the specificity of the antibody carrier molecule to its antigenic target is of extreme importance. In cancer immunotherapy, malignant cells often overexpress receptors belonging to the epidermal growth factor receptor (EGFR) family that is required for cell growth and survival (27). These are ideal antigen targets for ACs on malignant cells, which require high specificity and affinity to enable efficient payload delivery (28, 29). Conversely, latently infected cells are void of such antigenic markers which makes targeting them challenging. In this section, we will summarize the main targets of ACs on acutely and latently infected cells and discuss the advantages and disadvantages of each target. These targets include the HIV-1 envelope glycoprotein, HIV-1 coreceptors, and host membrane proteins (**Figure 2**).

### Envelope Glycoprotein

The HIV-1 envelope glycoprotein (Env) is the sole viral protein expressed on the membrane of viral particles as well as on HIV-1 infected cells with active viral replication and is, therefore, the primary target of antibody-based therapies (30). HIV-1 Env protein is expressed as a precursor gp160 molecule which is cleaved by furin into its gp41 and gp120 subunits. Three gp120 and gp41 subunits together form the HIV-1 Env trimer. Following CD4 receptor engagement, the metastable Env trimer undergoes conformational changes to orchestrate co-receptor binding and activation of the fusion machinery. Besides the expression of functional HIV-1 Env trimeric proteins on HIV-1 infected cells, other forms of Env are presented including uncleaved gp160, gp41 stumps, and aberrant trimers (30). The conformational plasticity and heterogeneity of HIV-1 Env can elicit antibody responses that are classified into three groups: binding but non-neutralizing, neutralizing but strain-specific, and bNAbs (31). The optimal carrier antibody for ACs, however, is not solely based on the broadest and most potent antibody that can neutralize most of the HIV-1 global strains. An efficient antibody carrier for ACs depends on the effector mechanism of its payload, the rate of internalization of the AC, and the subsequent intracellular routing of the carrier and payload. Because high antibody affinity and potency to HIV-1 Env does not guarantee antibodies to be the best AC carriers, the use of soluble CD4, non-neutralizing antibodies, and bNAbs were all explored as carriers for HIV-1 cure ACs (**Table 2**).

**Table 2:** Virus-directed antibody conjugates.

Antibody Conjugate	Target	Carrier	Payload	Payload Type	Reference
7B2-PIT	gp41	7B2 mAb	Porphyrin, IR700	Photosensitizer	(32)
rCD4-dgA	gp120	CD4 Receptor	Ricin A chain	Toxin	(33)
CD4 (178)-PE40	gp120	CD4 Receptor	Pseudomonas exotoxin	Toxin	(34)
907-RAC	gp120	907mAb	Ricin A chain	Toxin	(35)
0.5β-RAC	gp120	0.5β mAb	Ricin A chain	Toxin	(36)
0.5β-PE	gp120	0.5β mAb	Pseudomonas exotoxin	Toxin	(36)
DAB389-CD4	gp120	CD4 Receptor	Diptheria toxin	Toxin	(37)
3B3(Fv)-PE38	gp120	3B3 scFv	Pseudomonas exotoxin	Toxin	(38)
924-RAC	gp120	924mAb	Ricin A chain	Toxin	(39)
924-PAC	gp120	924mAb	Pulchellin A chain	Toxin	(40)
Anti-gp160-RAC	gp160	Anti-gp160 mAb	Ricin A chain	Toxin	(41)
7B2-RAC	gp41	7B2 mAb	Ricin A chain	Toxin	(39)
41.1-RAC	gp41	41.1 mAb	Ricin A chain	Toxin	(39)
7B2-PAC	gp41	7B2 mAb	Pulchellin A chain	Toxin	(40)
F105-P-*gag*	gp120	F105 Fab	*gag-*siRNA	siRNA	(42)
scFvCD7-9R-*vif*	*Vif*	Anti-CD7 scFv	*vif-*siRNA	siRNA	(43)
scFvCD7-9R-*tat*	*Tat*	Anti-CD7 scFv	*tat-*siRNA	siRNA	(43)
188-Re-246-D	gp41	246-D mAb	188-Re	Radionuclide	(44)
213-Bi-246-D	gp41	246-D mAb	213-Bi	Radionuclide	(44)
213-Bi-2556	gp41	2556 mAb	213-Bi	Radionuclide	(45)
225-Ac-2556	gp41	2556 mAb	225-Ac	Radionuclide	(46)
177-Lu-2556	gp41	2556 mAb	177-Lu	Radionuclide	(46)
Dox-P4/D10	gp120	P4/D10 mAb	Doxorubicin	Drug	(47)
e10-1074	V3-glycan	10-1074 bNAb	CCR5mim6	Inhibitor	(48)
ePGT121	V3-glycan	PGT121 bNAb	CCR5mim6	Inhibitor	(48)
ePGT122	V3-glycan	PGT122 bNAb	CCR5mim6	Inhibitor	(48)
ePGT128	V3-glycan	PGT128 bNAb	CCR5mim6	Inhibitor	(48)
e3BNC117	CD4bs	3BNC117	CCR5mim6	Inhibitor	(48)
ePGDM1400	V2-apex	PGDM1400	CCR5mim6	Inhibitor	(48)
ePGT151	gp120-gp41 interface	PGT151 bNAb	CCR5mim6	Inhibitor	(48)
e10E8	MPER	10E8 bNAb	CCR5mim6	Inhibitor	(48)
eE51	CD4i	E51 bNAb	CCR5mim6	Inhibitor	(48)
50-69-RAC	gp41	50-69 mAb	Ricin A chain	Toxin	(49)
gp120-brefeldin A	gp120	Anti-gp120 Polyclonal Ab	Brefeldin A	Drug	(50)
2F5-Cholesterol	gp120/membrane	2F5 bNAb	Cholesterol	Lipid	(51)
eCD4-Ig	gp120	Anti-CCR5 mAb	CCR5mim1	Small drug	(52)

### gp41

Early exploration of ACs for HIV-1 cure argued that gp41 might be a more suitable target than gp120 as gp120 exhibits significant heterogeneity and variability amongst various isolates of HIV (53). Moreover, gp120 shedding from the Env trimer releasing free monomeric gp120 could potentially bind anti-gp120 antibodies, further reducing the efficacy of gp120-directed antibodies (54). Gp41, besides acting as an anchor for gp120 contains highly conserved regions and is known to have a fusogenic potential, thus catalyzing membrane fusion (55). Furthermore, surface expression of the N-terminal half of gp41 on both the virion and infected cells render their susceptibility to immune surveillance (56). For these reasons, some of the earliest AC used mAb targeting gp41.

The rapid advances in mAb isolation and epitope mapping resulted in a family of anti-gp41 mAbs with defined epitopes (57). These anti-gp41 mAb directed towards the extracellular disulfide loop domain and heptad repeat region conjugated to toxins showed efficacy in killing infected T cell lines and monocytes (58, 59), and superior efficacy in both *in vitro* and *in vivo* mouse model compared to gp120-targeted ACs (39, 41). Moreover, the efficiency of gp41-directed ACs was further improved *via* the addition of soluble CD4 and virtually eliminated p24 production in a mouse model of infection, compared to a gp120-directed carrier requiring 15-times higher dosage with marginal effect (39).

The promising result with gp41-directed ACs also encouraged the construct of antibody-radionuclide conjugate directed towards gp41. Moreover, an important work by Tsukrov and colleagues showed that the low-level residual expression of gp41 on PBMCs isolated from ART-treated HIV-1 infected individuals is sufficient for antibody-radionuclide conjugate to deliver cytocidal radiation to infected cells making this epitope an attractive target for ACs (60).

### CD4-Binding Site

To facilitate entry of HIV-1, the trimeric HIV-1 Env binds receptor CD4 found on host cells. The CD4-binding site (CD4bs) found on HIV-1 gp120 is highly conserved and has a low degree of glycan masking (61) making it an attractive target for mAb-based therapy.

Early *in vitro* data demonstrated significant neutralization of HIV-1 infection in T-cell lines with recombinant soluble CD4 (sCD4), prompting the field to explore the potential of sCD4 for HIV-1 therapy (62). Consequently, sCD4 was explored as the first-ever cytotoxic carrier molecule for *Pseudomonas* exotoxin A (PE) to kill HIV-1 infected cells, which will be further discussed in a later section. Furthermore, the isolation and identification of CD4bs-directed bNAbs from HIV-1 infected individuals offered a variety of possible carriers for ACs. A more stable, affinity-matured, and broader 3B3 single-chain variable fragment (scFv) derivative of b12, a CD4bs-directed bNAb, was generated and exhibited enhanced activity as a carrier molecule for PE. The 3B3(Fv)-PE AC, solved the challenges faced by sCD4 as a carrier, such as toxicities and apparent enhancement of infection at a low sCD4 concentration (63). The availability of various bNAbs targeting this epitope, as well as the retained functionality of IgG subunits as a carrier makes this epitope a highly attractive target for ACs.

### Host Cellular Proteins

Alternatively, ACs can be designed to target host cellular markers of infection-susceptible cells, receptor and coreceptors used by HIV-1, and biomarkers expressed on latently infected cells (**Table 3**). AC strategies that rely on host factor targeting are not dependent on LRAs to reverse latency and induce viral protein expression, which is a major advantage compared to HIV-1 Env targeting strategies. This section will focus on various host factors targeted by ACs for HIV-1 cure (**Figure 2**).

**Table 3:** Host-directed antibody conjugates.

Antibody Conjugate	Target	Carrier	Payload	Payload Type	Reference
Anti-CD25-RAC	CD25	Anti-CD25 mAb	Ricin A chain	Toxin	(64)
Anti-CD4-PAP	CD4	Anti-CD4 mAb	Pokeweed antiviral protein	Toxin	(65)
Anti-CD45RO IT	CD45RO	Anti-CD45RO mAb	Ricin A chain	Toxin	(66)
Anti-CD5-PAP	CD5	Anti-CD5 mAb	Pokeweed antiviral protein	Toxin	(65)
Anti-CD7-PAP	CD7	Anti-CD7 mAb	Pokeweed antiviral protein	Toxin	(65)
DAB486-IL2	IL2R	IL2	Diphtheria toxin	Toxin	(67)
DAB389-IL-2	IL2R	IL2	Diphtheria toxin	Toxin	(68)
AL-57-PF-CCR5	LFA-1	AL-57 scFv	CCR5-siRNA	siRNA	(69)
CD7-9R-CD4-siRNA	CD4	Anti-CD7 scFv	CD4-siRNA	siRNA	(43)
CD7-9R-CCR5-siRNA	CCR5	Anti-CD7	CCR5-siRNA	siRNA	(43)
4M5.3-3X4	CXCR4	Anti-CXCR4 scFv	*tat-siRNA*	siRNA	(70)
CCR5mAb-FI	CCR5	Anti-CCR5 mAb	T-2635	Small drug	(71)
38C2-Apalviroc	CCR5	38C2 cpAb	Aplaviroc	Small drug	(72)
cP4/D10-T20	gp120	P4/D10 mAb	Enfuvirtide	Small drug	(73)
38C2-BMS-488043	CCR5	38C2 cpAb	BMS-488043	Small drug	(74)
38C2-Maraviroc	CCR5	38C2 cpAb	Maraviroc	Small drug	(75)

Reasonably, the first host factor targeted by ACs is the HIV-1 entry receptor, CD4. CD4 is expressed on the surface of T cells, monocytes, macrophages, and dendritic cells (76). An anti-CD4 mAb conjugated with toxin exhibited selectivity and efficacy in eliminating HIV-1 production in activated CD4+ T cells from an infected individual *in vitro.* Since HIV-1 mainly infects CD4+ T cells, additional T cell markers were also targeted by the conjugation of toxins and small-interfering RNA (siRNAs) to anti-CD5 and anti-CD7 mAb (43, 65, 77). Additionally, markers of T cell activation such as IL2 receptor, CD25 (or IL-2 receptor α-chain), and CD45RO, were also the target of several ACs, which aims at targeting only activated T cells, while sparing quiescent T cells (43, 65).

Coreceptor targeting emerged as a central theme of subsequent ACs for HIV-1 cure. CCR5 and CXCR4 are the coreceptors required for HIV-1 viral entry and are widely expressed on immune cells including CD4+ T-cells. The choice of coreceptor generally determines viral tropism with CCR5, and CXCR4 usage for R5, and X4 viruses, respectively (64, 66–68, 78). CXCR4 constitutes a highly attractive target for ACs because this receptor is efficiently and rapidly internalized after interaction with its natural ligand, SDF-1α (79). CXCR4 is therefore an ideal target for ACs with payloads requiring internalization to function. However, to date, only one AC construct targeted this receptor for the delivery of an anti-*tat* siRNA conjugated to a CXCR4 nanobody (70, 80).

Coreceptor targeting favored CCR5 over CXCR4 mainly because R5 tropic viruses are predominantly involved in the early viral transmission of infection, predominate in the asymptomatic stage of infection, and persist throughout all stages of the disease (81, 82). In comparison, the emergence of X4 tropic or dual tropic R5X4 viruses tends to occur at a later stage of infection to only about 50% of progressing patients (83, 84). The predominance of R5 tropic viruses during infection explains the preference of targeting the CCR5 coreceptor by ACs. Furthermore, the availability of structural data of CCR5-Env interaction further fueled the development of many CCR5-targeting small molecules and mAbs (85–88). One class of CCR5-targeting molecules is CCR5 sulfopeptide mimetics that simulate CCR5-Env interaction (71, 89–91). These mimetic peptides were later used by several ACs discussed in a later section. Overall, the targeting of HIV-1 coreceptors is an attractive host factor target for ACs as they are implicated in viral entry and transmission.

Lastly, lymphocyte function-associated antigen-1 (LFA-1) was also the target of an AC aiming at preventing the cell-cell spread of HIV-1. LFA-1 is an integrin expressed on all lymphocytes and is involved in emigration and adhesion processes (69). Additionally, LFA-1 expression increases during HIV-1 infection and is also implicated in the formation of virological synapse for HIV-1 propagation (92). It was therefore hypothesized that an anti-LFA-1 mAb could be used therapeutically against HIV-1 infection. Early studies indeed showed that an anti-LFA-1 mAb could reduce HIV-1 RNA in HIV-1 infected individuals (93), and more recently it was found to inhibit the cell-cell spread of infection (94). Cell-cell transmission of HIV-1 plays an important role in the propagation of infection in addition to cell-free infection of target cells, with some reporting that cell-cell infection could be the main route of HIV-1 infection (95–97). Although the efficiency of cell-cell *vs* cell-free propagation of HIV-1 is still under debate, the availability of mAb that can inhibit cell-cell spread could be an attractive carrier for ACs. Therefore, host factors such as LFA-1 that are involved in the formation of virological synapse and cell-cell propagation are another important target for HIV-1 cure ACs.

Ultimately, the most convenient host factor targets for HIV-1 ACs are latency surface biomarkers only expressed on infected cells. The discovery of such latency biomarkers is an active area of research. Enrichment of certain surface markers such as CD2, CD20, CD30 and immune checkpoint inhibitors on latently infected cells are currently being explored as potential host biomarkers of latency (98, 99). To further highlight the substantial challenge of finding such biomarker, the discovery of CD32a as a marker of latency associated with enrichment of HIV DNA (100), has been challenged by subsequent studies (101–103). However, while the discovery of such biomarkers is underway, ACs can overcome this limitation by designing constructs with which the payload confers the selectivity against viral factors while remaining inert in uninfected cells. Therefore, host-targeting ACs could be an attractive method for conjugation with oligonucleotide or small drugs that are highly HIV-1 genome-specific.

## Antibody Conjugates for HIV-1 Cure

The approval of zidovudine in 1987 as the first drug for HIV-1 ART revealed the apparent need for an alternative curative agent to address the inability of ART to fully eradicate HIV-1. ACs are an attractive complement to ART because of their high selectivity and targeted delivery of cytotoxic and immune regulating payload. Some of the advantages of ACs over their unconjugated components are increased therapeutic index of the payload, reduction in off-target toxicities, increased circulation half-life, and selectivity accomplished by the carrier molecule to its antigen. Furthermore, once a certain threshold of binding is achieved, the use of high-affinity antibodies is not as crucial for efficacy as unconjugated antibodies (104). ACs can be further modified for internalization capacity, intracellular routing, controlled payload release, drug-to-antibody ratio, and linker chemistries, which is beyond the scope of this review.

In this section, the diversity of ACs for HIV-1 cure is explored. Special attention is given to mAb-based carriers for molecular payload; therefore, this review excludes other targeted approaches such as immunoliposomes, nanoparticles, dendrimers, aptamers, etc. Although not a mAb carrier, the use of sCD4 as a carrier of a cytotoxic payload is also discussed in this review as the use of this construct marked the origination of a highly targeted therapeutic approach for HIV-1 cure.

Currently, the AC landscape for HIV-1 cure is diverse and depending on the payload used the naming conventions are not standardized. This naming convention phenomenon mainly occurred because of the asynchronous development of ACs in cancer immunotherapy. In this review, a naming convention focusing on the payload is used (**Table 1**). This aims to consolidate the field of using antibodies as the primary carrier of cytotoxic and immune regulating payload. Therefore, antibody conjugation of toxins herein referred to as antibody-toxin conjugates; antibody conjugation of radionuclide, herein referred to as antibody-radionuclide conjugates; antibody conjugation of drugs/small molecules, herein referred to as antibody-drug conjugates; antibody conjugation of an oligonucleotide, herein referred to as antibody-oligonucleotide conjugates, and other novel payloads discussed herein will follow a similar naming convention.

**Figure 3:**
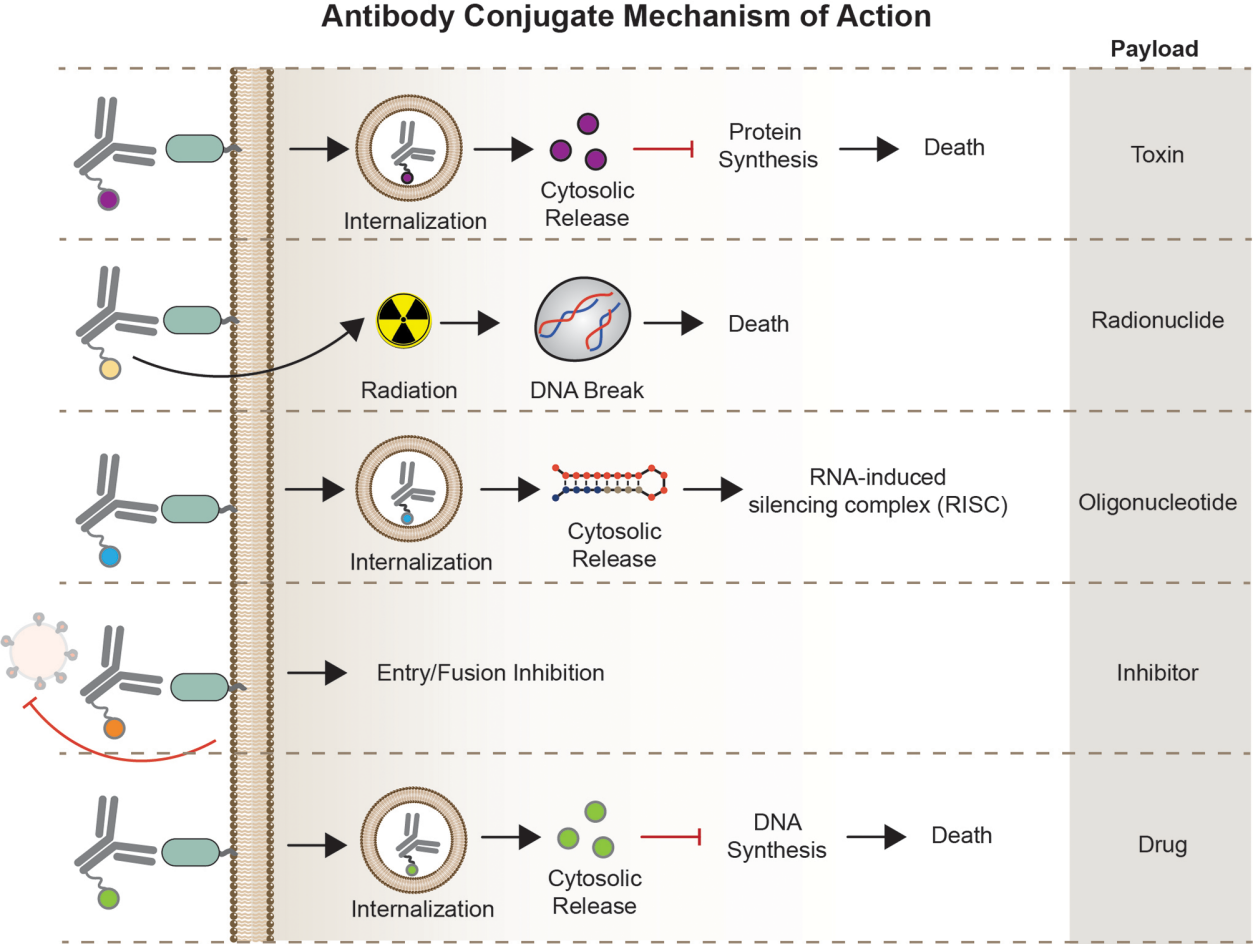
Mechanism of Action. Antibody conjugates execute different mechanism of action depending on the carrier molecule, antigen, internalization requirement, and effector function of the payload. Antibody-toxin binds to target epitopes, internalized, followed by escape of toxin to the cytosol to halt protein synthesis. Antibody-radionuclide binds to the receptor to deliver ionizing radiation to the cell inducing DNA breaks. Antibody-oligonucleotide binds to its receptor, internalized, and bind complementary sequences in the cytosol for gene silencing. Antibody-drugs can act either internally or non-internally depending on the effector molecule – fusion inhibitor binds to a receptor or viral particle to inhibit entry, some classes of cytotoxic compounds act by intercalating to cell’s DNA inducing DNA/RNA synthesis.

Overall, this section aims to describe each AC type focusing on their unique structure, mode of action (**Figure 3**), potential therapeutic use, as well as challenges in the bigger HIV-1 cure landscape.

### Toxins

The antibody-toxin conjugate is the fusion of toxin payload to a carrier antibody molecule. Antibody-toxin is more commonly known in the field of cancer immunotherapy as an immunotoxin. However, immunotoxins can be characterized as the fusion of toxins to other carrier molecules such as growth factors, cytokines, or soluble ligands. For this review, antibody-toxin is used when the carrier molecule is an antibody.

Typically, a toxin contains a cell-binding domain, a translocation domain, and a catalytic effector domain responsible for cell death. An antibody-toxin replaces the cell-binding domain of toxins with a mAb carrier to provide selectivity, retaining the translocation and catalytic effector domains for functionality. To be effective, antibody-toxins must bind to their antigen, rapidly internalize, and translocated to the cytosol where it can catalytically disrupt protein synthesis leading to cell death. Toxins used in HIV-1 ACs are derived from bacteria such as PE, Diphtheria toxin (DT), and anthrax toxin, and plants such as pokeweed, and ricin. These toxins are an ideal payload for ACs due to the ease of recombination, high expression and yields, potency, and relatively low toxicity compared to other toxins (105).

The first-generation toxin conjugates for HIV-1 were developed in the late 1980s. Soluble CD4 was the first carrier molecule for PE, and ricin A chain (RAC) producing CD4-PE40 and rCD4-dgA constructs, respectively. These toxin conjugates showed high efficacy in targeting gp120-expressing cells and inhibiting HIV-1 protein synthesis in both acute and chronic HIV-1 T cell line models (34, 58). PE and RAC, upon internalization, interrupt HIV-1 protein synthesis by ADP-ribosylation and the inactivation of the 60S ribosomal subunit, respectively. Toxin DT conjugated to sCD4, DAB389CD4, also exhibited highly efficient killing of HIV-infected cell lines *in vitro*, as well PBMCs from HIV-seropositive patients, and displayed no apparent toxicity. However, in both HIV-infected cell lines and PBMCs, DAB389CD4 resistant strains emerged (37, 106). DT conjugation to IL-2 (DAB486-IL2) also exhibited potency in eliminating HIV-1 infected cells in a mixed culture of infected and uninfected T cells detected by the inhibition of HIV-1 protein and RNA production (67).

The early efficacy in *in vitro* characterization of CD4-PE40 in cell line models pushed the field to extensively evaluate this toxin conjugate. The construct exhibited favorable potency with half-maximal inhibitory concentration (IC_50_) <0.1 nM in cell culture, efficacy in monocytes and macrophages, little toxicity against uninfected cells, and efficacy in primary isolates of HIV-1 (107, 108). Additionally, CD4-PE40 demonstrated synergy when combined with ART (109), and was well tolerated in rhesus macaques (110). However, in a human phase I clinical study, a single low dose of CD4-PE40 at 15 µg/kg resulted in hepatic toxicity with no apparent antiviral activity (110). The same results were attained in a human phase III multi- and dose-escalating treatment (111). Both human trials resulted in dose-related toxicities, induction of anti-drug antibodies (ADA), and limited circulation half-life of only 3 hours.

The discouraging results of the first-generation toxin conjugates based on CD4 carriers prompted the exploration of new carriers that could offer increased circulation half-life, reduced hepatotoxicity, and prevents potential involvement of sCD4 in the release of gp120 on the virion or cell surface (39, 112). The use of mAb carriers could address the shortcomings of CD4 as toxin carriers, which led to the development of the second-generation toxin conjugates to mAb targeting HIV-1 Env. The chemical conjugation of RAC to 50-69 and 907 mAbs, both targeting gp41 showed efficacy in infected cell lines with IC_50_ in the nM range (35, 58). Furthermore, 924-RAC, targeting gp41, exhibited superior efficacy and addressed most of the shortcomings of CD4-PE40 and showed increased potency when combined with CD4-Ig in an *in vivo* mouse model of infection, and decreased susceptibility to blocking effects from anti-HIV antibodies found in the serum of HIV-infected patients (39, 113). Therefore, the use of antibodies as carriers for toxins showed a more favorable outcome both in potency and pharmacokinetics of the construct in comparison to sCD4.

Antibody-toxin conjugates based on PAP conjugated onto antibodies targeting CD4, CD5, and CD7 showed pM efficacy in the inhibition of HIV-1 replication in primary CD4+ T cells (65). Moreover, anti-CD4-PAP was found effective at inhibiting HIV-1 production for several weeks in an *ex vivo* samples of activated replicating CD4+ T cells, as well as in clinical HIV-1 isolates *in vitro* and demonstrated superior anti-HIV-1 activity compared to zidovudine treatment, and also exhibited efficacy against zidovudine-resistant viruses (65, 114). Subsequently, attempts were made to specifically target the latent reservoirs *via* antibody-toxin conjugates. Antibody-toxin directed to CD25 and CD45RO aims at targeting activated and latent cells, respectively. These antibody-toxins proved to be effective in eliminating replication-competent HIV-1 infected PBMCs as well as *ex vivo* treatment of CD4+ T cells from HIV-infected individuals (64, 66, 115).

Currently, antibody-toxins are taking advantage of the recent advancement in bNAb discovery, antibody engineering, and ART complementation to further increase their therapeutic value. A mAb carrier based on b12 bNAb subunit, 3B3 scFv, fused to PE38 (a truncated version of PE40), exhibited enhanced therapeutic attributes than the previous generation of toxin conjugates. 3B3(Fv)-PE38 retained broad reactivity against HIV-1 isolates, enhanced cytotoxicity in transfected cell line models compared to CD4-PE40, and suppressed viral load in an *in vivo* mouse model. Further characterization of 3B3(Fv)PE38 also showed the ability to block primary HIV-1 isolates in both PBMCs and monocyte-derived macrophages, and encouragingly, showed no apparent *in vivo* hepatotoxicity in rhesus macaques that were previously observed with CD4-PE40 (38, 116–118).

In the context of HIV-1 treatment, antibody-toxins are likely to be most effective when combined with ART. This has already been indicated in several studies showing that antibody-toxin in combination with ART, is more effective in blocking infection than when used alone *in vitro* and *in vivo*, as well as a sustained delay in viral rebound than combination ART treatment alone after cessation (109, 116, 119).

Despite decades of antibody-toxin conjugate research for HIV-1 cure, no such constructs are approved therapeutically. ART and antibody-toxins were developed side-by-side in the early days of the AIDS epidemic with ART significantly changing the course of the disease. Antibody-toxins for HIV-1 cure, therefore, warrants a second look because of the following advantages that they offer; 1) antibody-toxin can target actively and latently infected cells where current ART is ineffective, 2) antibody-toxin have reduced toxicities and improved half-life compared to CD4-PE40, 3) a variety of antibodies can be used as next-generation carriers with broad reactivity against HIV-1 strains, and finally, 4) antibody-conjugates are proven safe and effective in the field of cancer immunotherapy.

### Radionuclides

Antibody-radionuclide is the conjugation of radioisotopes to a carrier mAb to deliver lethal doses of ionizing radiation to cells. In the field of cancer immunotherapy, antibody-radionuclide is also referred to as radioimmunotherapy. The main mode of action of antibody-radionuclide is the radiation-induced cell death *via* double-stranded DNA breaks or the formation of reactive oxygen species (ROS) upon engagement with its antigen. The FDA approval of Ibritumomab tiuxetan (Zevalin) for the treatment of non-Hodgkin’s lymphoma in 2002 marked the first clinical use of RIT. Zevalin uses an anti-CD20 mAb carrying a radioactive ^90^Yttrium and is administered as a single dose.

Dadachova and colleagues reported the first proof-of-concept construct of antibody-radionuclide for HIV-1 with the conjugation of bismuth 213 (^213^Bi) and rhenium 188 (^188^Re) to anti-gp120 and anti-gp41 antibodies. These antibody-radionuclides selectively killed chronically HIV-1 infected cells and acutely infected PBMCs *in vitro*. The degree of cytotoxicity depends on the energy and half-life of the conjugated radionuclide. Gp41-^188^Re exhibited superior potency in an *in vivo* mouse model due to the longer physical half-life of ^188^Re (t_1/2_ = 16.9 h), enabling adequately access to infected cells in the circulation in comparison to ^213^Bi (_t1/2_ = 46 m) which loses its radioactivity at a relatively shorter time (44).

To further elucidate the contribution of the radionuclide’s physical properties for its efficacy, different radionuclides were conjugated to the same antibody carrier. Garg and colleagues conjugated an anti-gp41 mAb 2556 with ^213^Bi (_t1/2_ = 46 min, alpha radionuclide), and two radionuclides with a much longer half-life, ^225^Actinium (^225^Ac, _t1/2_ = 9.9 days, alpha radionuclide), and a beta emitter ^177^Lutetium (^77^Lu, _t1/2_ = 6.7 days). Three days post-treatment, both ^213^Bi, and ^177^Lu antibody-radionuclides significantly killed PBMCs infected with HIV-1_p49.5_ *in vitro*, while ^225^Ac antibody-radionuclide exhibited minimal potency. However, at 7 days post-treatment, all three antibody-radionuclides showed a significant reduction of p24 levels compared to an anti-RSV mAb control (46). Similar results were observed in the infected CD14^+^CD16^+^ monocyte, which is known to play a role in HIV-1 neuropathogenesis.

Antibody-radionuclides were observed to be more potent in killing monocytes than other cell populations. This led McFarrren and colleagues to test their efficacy in targeting HIV-1 reservoirs in the central nervous system (CNS). Using an *in vitro* blood-brain barrier (BBB) model the group added 2556-^213^Bi AC in PBMCs and monocytes that transmigrated across the BBB. This AC induced increased apoptosis with 30% of infected PBMCs and 60% of infected monocytes killed. However, a high level of nonspecific apoptosis of uninfected monocytes was observed, in some conditions reaching up to 80% bystander killing. This could be due to the high concentration of radiation in the sample, or cell crowding in the BBB *in vitro* model causing the crossfire effect (45).

Finally, the efficacy of antibody-radionuclide complemented with ART was evaluated by Tsukrov and colleagues. Both in an *ex vivo* infection model and ART-treated PBMCs from HIV-1 infected individuals, the treatment with 2556-^213^Bi demonstrated no negative effect and exhibited similar potency as ART-naïve PBMCs (60).

Antibody-radionuclide conjugates are a valuable addition to cancer immunotherapy, for HIV-1 cure, however, a different set of challenges must be overcome. Both approved antibody-radionuclide conjugates for cancer immunotherapy are not used as first-line treatment and are most widely applied to most radiosensitive tumors, such as leukemias and lymphomas. Therefore, the utility of antibody-radionuclide conjugates as a therapeutic intervention in the context of HIV-1 infection must be further explored. As with antibody-toxin conjugates, antibody-radionuclide conjugates are more likely to be effective in combination treatment with ART, therefore the optimal timing of treatment in acute *vs* chronic HIV-1 infection, as well as its effect during the immunocompromised status of infection must be investigated. Next, the sensitivity and dosimetry profiles of actively and latently infected cells for radiation, as well as HIV-1 sanctuary sites must be established. Finally, since HIV-1 persistence in macaque models might not recapitulate HIV-1 infection in humans, efficacy studies will be best performed in humans, which could add more complexities in testing its efficacy and safety in humans.

### Small Drugs

Antibody-drug conjugate is a targeted drug delivery approach characterized by the fusion of a small molecule drug to a carrier mAb. In the cancer immunotherapy field, antibody-drug conjugates, or ADCs, are commonly designed with the conjugation of a potent cytotoxic agent that can induce cell death. However, antibody-drug conjugates for HIV-1, as described in this review, also include conjugation of small molecules and peptides that can block infection and are not apoptotic agents.

Repurposing of approved drugs proved to be beneficial in the field of HIV-1 cure. Zidovudine, originally intended for cancer treatment, was repurposed for HIV-1 treatment and approved by the FDA as the first-ever treatment available for HIV-1. Hence, the pioneering antibody-drugs for HIV-1 conjugated doxorubicin, a cancer drug, to a murine anti-gp120 mAb, P4/D10. The P4/D10-doxorubicin conjugate exhibited protection in mice after challenge with an 8-fold less concentration compared to the unconjugated mAb (47). This construct was further optimized using a humanized P4/D10 mAb conjugated to approved HIV-1 drug enfuvirtide to reduce potential immunogenicity of the murine P4/D10. The hP4/D10-enfuvirtide conjugate exhibited improved potency in both pseudovirus and cell-spread assays (73).

These studies demonstrated that conjugation of drugs with different modes of actions can be effective when designed as ACs. Doxorubicin and enfuvirtide are two small drugs that inhibit nucleic acid synthesis, and viral entry, respectively. Additionally, another intracellularly-acting payload, brefeldin A, inhibiting ER-Golgi protein trafficking, was also shown to be an effective payload for ACs (50).

ACs with small drugs later focused on increasing potency by blocking cell entry and fusion. Particularly, CCR5 antagonism was favored. A promising early study by Ji and colleagues showed synergistic potential *in vitro* when an anti-CCR5 mAb was combined with a CCR5 small molecule antagonist targeting non-overlapping epitopes (88). Encouraged by this result, Kopetzi and colleagues designed a bifunctional AC consisting of an anti-CCR5 mAb covalently linked to T-2635, a fusion inhibitor. This construct exhibited efficient blocking of HIV-1 Env-mediated fusion but showed viral tropism dependency with its inability to prevent X4-tropic infection in PBMCs (71).

Alternatively, several groups explored the conjugation of CCR5 antagonist to a chemically programmed antibody (cpAb) 38C2 (120). However, this construct is different than the traditional AC since the CCR5 antagonist itself provides the targeting and effector function, while the antibody carrier mainly functions to extend the pharmacokinetic profile of CCR5 antagonists (**Figure 1C**). The 38C2 cpAb can form a site-selective conjugation with *N-acyl*-*β-*lactam derivatives of drugs and small molecules. Explored here includes *β-*lactam derivatives of BMS-378806, BMS-488043, Apalviroc, and Maraviroc (72, 74, 75). This conjugation technique dramatically extended the pharmacokinetic profiles of the attached molecule which increased serum stability compared to its unconjugated counterpart, however, this technique only exhibited minimal improvement in neutralization potency compared to the parental molecule.

Improvement in potency was later observed when Gardner and colleagues designed eCD4-Ig, which is a fusion of a CCR5 mimetic peptide, mim1, to CD4-Ig. This construct can simultaneously bind to CD4- and coreceptor-binding site on HIV-1 Env, and was found to have an increased breadth and similar potencies as bNAbs (48, 52). Subsequent variants of eCD4-Ig were designed with improved versions of mim1. These eCD4-Ig variants exhibited cooperative avidity to Env, improved potency and breadth compared to bNAbs tested, ability to neutralize neutralization-resistant strain of HIV-1, and showed protection in rhesus macaques after viral challenge (52, 121, 122). These encouraging results prompted the conjugation of the CCR5 mimetic to bNAbs belonging to different classes. When conjugated to the C-terminus of bNAbs, mim6, exhibited improved potency of all classes of bNAbs against HIV-1 isolates. However, this is only true to HIV-1 isolates that were previously sensitive to mim6, suggesting possible bNAb epitope dependency. Indeed, the importance of bNAb carriers to potentiate improved potency of conjugated mim6 was exhibited by its conjugation to V3-targeting bNAbs. V3-targeting bNAbs conjugated with mim6 displayed a 2-fold increase in potency even against mim6-resistant HIV-1 isolates tested (48).

Antibody-drug development for HIV-1 cure is slow, in comparison to cancer immunotherapy, and more preclinical data is needed to evaluate its potential as a therapy for HIV-1. To date, only one study showed the possible cooperative potential of antibody-drug as a complement to ART, which was investigated in the late 1990s. An anti-gp120 mAb-brefeldin A conjugate exhibited efficacy in a chronic cell line model of infection as well as infected PBMCs when used alone. However, antibody-brefeldin A combined with zidovudine resulted in a remarkable 90% reduction of virus production in an IC_50_ in the nM range, potency in zidovudine-resistant virus strain, and low toxicity against uninfected cells (50).

In a recent patent application, the repurposing of commonly used anti-cancer payloads in cancer antibody-drug was outlined. An HIV-1 antibody-drug was designed with the conjugation of a bNAb to monomethyl auristatin E (MMAE), a synthetic antineoplastic agent commonly used in cancer antibody-drug conjugates. The AC vc-MMAE-NIH45-46 G54W, which is the conjugation of NHI45-46 G54W bNAb to MMAE, could be further explored and highlights the potential application of various cytotoxic drugs not originally intended for HIV-1 cure as a payload for HIV-1 AC (123).

Overall, the success of antibody-drug conjugates in cancer immunotherapy can potentially be mirrored and translated for an HIV-1 cure. The early preclinical successes in the conjugation of HIV-1 drugs, small drugs, and peptide inhibitors to mAbs described herein display a great deal of promise. To move forward, future development of antibody-drugs for HIV-1 should also explore the potential efficacy of conjugating other HIV-1 inhibitors blocking various stages of the viral replication cycle besides entry and fusion inhibition. More importantly, future antibody-drugs should explore drug-drug interaction under current ART and establish its cooperative tendencies to ART, effectiveness alongside LRA, and finally, monitor any potential development of resistance to the drug or AC itself.

### Oligonucleotide

The conjugation of an oligonucleotide to a mAb herein referred to as antibody-oligonucleotide conjugate, describes the use of mAb as the carrier molecule to RNA, DNA, or synthetic oligonucleotide that often exerts a variety of functions. A variety of engineered oligonucleotides based on their functionality has been described. This includes oligonucleotide involved in targeting (aptamers), gene expression regulation (miRNA), gene silencing (antisense oligonucleotide or ASO, and siRNA) (124).

Twenty years ago, Elbashir and colleagues published the hallmark proof-of-principle experiment demonstrating the use of siRNA to knockdown a specific gene in a mammalian system (125). This discovery led to an increased interest in RNA interference (RNAi)-based technologies leading to the first siRNA-based drug approved by the FDA in 2018, Onpattro, to treat polyneuropathy in people with hereditary transthyretin-mediated amyloidosis (126).

RNAi technology uses 19-23 base pair (bp) RNA duplexes that intervene post-translationally triggering cellular degradation of cognate messenger RNA (mRNA). RNAi can silence a gene with high specificity and can virtually target any gene. The potential of RNAi is challenged by stability, biodistribution, delivery to target cells, and *in vivo* delivery across the cell membrane to the cytoplasm where the mRNA is located (127).

In 2002, hallmark papers showed the capacity of RNAi technology to silence HIV-1 replication and production *via* the use of synthetic siRNA. Researchers showed the efficacy of siRNA to target both the pre- and post-integration RNA of the HIV replication cycle by targeting HIV genes [*vif, nef, tat, rev, p24*, and long terminal repeat (LTR)] (128, 129). Additional studies by Novina and colleagues showed the potential use of siRNA in targeting host cellular factors by designing siRNA specific for human CD4, which exhibited a reduction of viral entry and viral production, as well as siRNA targeting HIV-1 coreceptors CXCR4 and CCR5 (130).

Various methods are actively being developed to solve the delivery problem with oligonucleotides including chemical modifications, and conjugation of siRNA to targeting moiety. However, issues of rapid clearance (siRNA-peptide conjugation), potential immunogenicity (liposome, PEGylation), and increased off-targeting and toxicity (high dosage) remain a problem (127, 131). The use of mAb as a siRNA carrier could address such limitations by enabling a cell-targeted approach, reduction of siRNA payload, limiting nonspecific silencing, and minimizing related toxicities. In this section, the current state of antibody-oligonucleotide for HIV-1 cure is detailed.

The first proof-of-principle antibody-oligonucleotide conjugate for HIV-1 cure was designed by Song and colleagues with an anti-*gag* siRNA fused to the F105 Fab antibody fragment. The AC was engineered from a bicistronic plasmid encoding the Fab fragments of the F105 heavy- and light-chains with protamine fused at the C-terminus (**Figure 1B**). Protamine can form a complex with oligonucleotides, which bypasses the need for covalent coupling. F105-P *gag* siRNA induced gene silencing of targeted cells and inhibition of HIV replication in HIV-infected primary T cells, as well as in an *in vivo* mouse model (42).

Subsequently, antibody-oligonucleotide conjugates displayed efficacy when tested in an *in vivo* humanized mouse model. Using an scFv targeting CD7, a pan-T cell receptor, anti-CCR5 siRNA, and a combination of siRNAs targeting viral genes *vif* and *tat* were conjugated *via* the addition of nine arginine residues (9R) to scFv which facilitated the conjugation of siRNA through charge interaction. Intravenous injection of scFvCD7-9R siRNA halted CD4+ T cell decline and reduced viral loads in comparison to controls in a humanized mouse model (43). Using another host-expressed protein as a target, Peer and colleagues designed AL-57-PF-CCR5, an scFv targeting LFA-1 conjugated with an anti-CCR5 siRNA. This construct showed effective reduction of mRNA expression in memory T cells expressing LFA-1, attenuating CCR5 expression in activated T cells (69).

**Figure 4:**
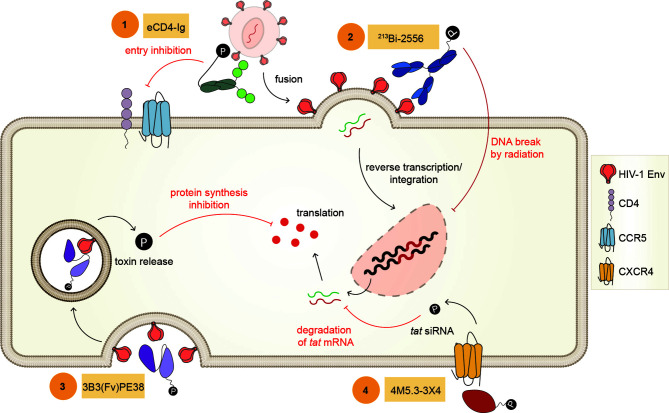
Example of Antibody Conjugates. Representative antibody conjugates described in this review (1) eCD4-Ig is the conjugation of CD4 to a CCR5 mimetic peptide inhibiting viral fusion and entry, (2) ^213^Bi-2556 is an antibody-radionuclide with ^213^Bi fused to an antibody targeting gp41, this conjugate delivers cytocidal radiation upon binding inducing DNA break, (3) 3B3(Fv)PE-38 is an antibody-toxin constructed with the scFv subunit of b12 bNAb conjugated to PE toxin and functions by inhibiting protein synthesis upon internalization, (4) 4M5.3-3X4 is an antibody-oligonucleotide designed with a nanobody targeting CXCR4 conjugated with an anti-*tat* siRNA, inhibiting Tat-driven transcription.

It took over a decade for the next antibody-oligonucleotide construct to be published. Using a nanobody against CXCR4, an anti-*tat* siRNA was conjugated. The construct 4M5.3X4 efficiently delivered anti-*tat* siRNA to CXCR4+ cell lines as well as human primary T cells, and abolished Tat-driven HIV transcription (70). This construct also marked the first time a nanobody was used as a carrier for HIV-1 AC (**Figure 4**).

The field of antibody-oligonucleotide conjugates for HIV-1 cure is at the early phases of exploration. Although siRNA must escape to the cytoplasm to exert its gene-silencing function, the trafficking pathway of the constructs described herein remains to be understood. Additionally, for antibody-oligonucleotide conjugates to be effective, timing and durability are crucial. It is still not known whether transient treatment with antibody-oligonucleotide conjugates could offer sustained silencing of targets that is therapeutic in HIV-1 infected individuals. Furthermore, more preclinical data is needed to determine whether endogenous siRNA could potentially interfere with antibody-oligonucleotide, or whether off-target effects will be an issue.

### Novel Payloads

Innovative conjugation techniques are enabling the development of novel payloads for ACs. One such example is the conjugation of photosensitizers (PS) to antibodies. Antibody-photosensitizer conjugates, also known as photoimmunotherapy (PIT), is a targeted photodynamic therapy designed with a conjugated PS to a mAb. PIT is proposed to be minimally invasive and safer than conjugated toxins or radionuclide, reducing possible immunogenicity and crossfire effects, respectively. Conventional PS treatment using light called photodynamic therapy (PDT), destroys cells nonspecifically when exposed to light through the production of ROS. The inclusion of mAb as the targeting molecule thus provides selectivity. Sadraeian and colleagues showed the first proof-of-principle use of antibody-photosensitizer conjugate for HIV-1. Using 7B2 anti-gp41 carrier antibody, a cationic porphyrin and anionic IR700 PS were conjugated and tested for cytotoxicity in an Env-transfected cell line. The addition of both porphyrin- and IR700-conjugated 7B2 exhibited an increase in ROS levels after irradiation compared to untransfected controls. Following the increase in ROS level, cell death was observed. Although the adaptation of PDT to HIV-1 described in this study is preliminary, this approach is a welcomed addition to the increasing lineup of ACs for HIV-1 cure. Further studies are warranted to fully elucidate the potential of antibody-photosensitizer in the field of HIV-1 cure, especially its effectiveness in non-solid tumors, such as HIV-1, considering that the main restriction of this approach is the constraint of light penetration.

The conjugation of cholesterol to mAb was also explored. When cholesterol was conjugated outside the paratope of the membrane-proximal external region (MPER)-class of bNAbs, Lacek and colleagues found that the neutralization activity of the bNAbs was dramatically increased. This antiviral potentiation is due to the increased interaction between cholesterol and the lipid raft domains on the membrane. The antibody-cholesterol conjugate was also shown to rescue the activity of a mutated version of an MPER bNAb with abolished interaction to the viral membrane, with the cholesterol moiety mimicking the interaction. Therefore, cholesterol conjugation can be a valuable tool in increasing the antiviral activity of bNAbs. Since the conjugation can be made at several positions on the mAb and occurs outside the antibody paratope, it can complement various affinity maturation strategies (51)

Lastly, in recent years there has been an interest in the use of Toll-like receptor (TLR) agonists in the context of HIV-1 cure. The engagement of TLRs and their ligands can lead to activation of the innate immune response and priming of the adaptive immune system. Therefore, TLR agonists can play a dual role in HIV-1 by acting as an LRA and an immune regulator (132). In the HIV-1 cure context, Borducchi and colleagues showed that a combination treatment of a TLR7 agonist (GS-9620) and PGT121 delayed viral rebound following ART interruption in rhesus macaques. Moreover, 5/11 treated animals did not result in a viral rebound following ART interruption (133). These results, therefore, provide a good rationale for conjugating TLR agonists to mAb. An antibody-TLR agonist conjugate can directly target immune activation to latently infected cells, activate effector cells, and potentiate bNAb function by immediate recognition of reactivated cells. The conjugation of TLR agonists to mAb is already being explored in cancer (134).

## Antigen Binding and Internalization

**Figure 5:**
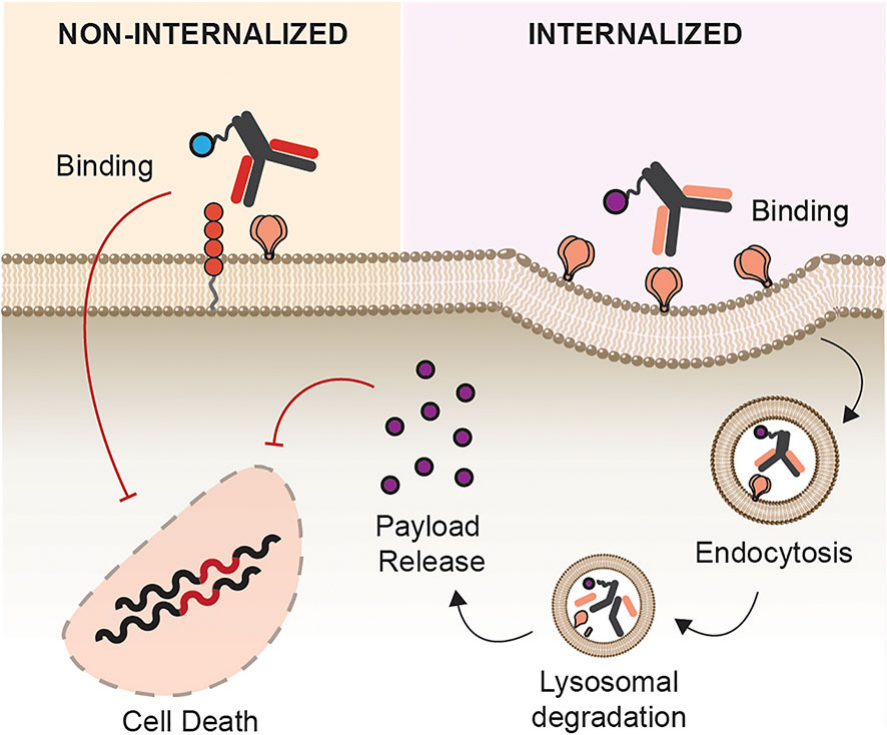
Site of Action. Antibody conjugates can act either intracellularly or intercellularly upon antigen binding. Toxins, siRNA, and inhibitors of DNA synthesis require internalization as they are only functional upon cytosolic release. Payloads such radionuclide, entry inhibitors, and photosensitizers are functional without internalization.

Another essential factor in the efficacy of ACs, besides antigen specificity and payload efficacy, is the internalization requirement of ACs (**Figure 5**). ACs described herein showed different requirements for cellular uptake, internalization, and intracellular routing. While radionuclides were functional upon mere binding to its antigen, certain payloads require internalization, effective linker cleavage, and lysosomal escape to be functional such as toxins, oligonucleotide, and small drugs. In cancer immunotherapy, alongside antigen density, the varying rate of internalization after AC engagement is directly linked to its efficacy (135). It is therefore not a surprise that payloads that act intracellularly were most effective when conjugated with a rapidly internalized receptor, and antibodies binding to a more proximal epitope to the plasma membrane (136), which is the case of siRNAs and toxins, respectively (59, 70).

The internalization kinetics, therefore, must be considered when designing the next-generation ACs. This parameter has not been fully exploited by ACs described herein. For example, an internalized radionuclide in combination with a radionuclide having a shorter range of radiation could decrease the potential crossfire effect. Additionally, a combination of a small drug payload targeting intracellular viral proteins (reverse transcriptase, integrase, protease) could be effective when endocytosed and released into the cytoplasm.

## Safety and Immunogenicity

The disappointing termination of CD4-PE40 in human clinical trials, the only AC for HIV-1 cure to date, because of safety concerns highlights the need to improve both the efficacy and safety in future AC development. The diversity of ACs for HIV-1, including its choice of antigen, carrier molecule, types of payloads, mode of action, and linker chemistries, complicates the generalization of the safety profile of each AC category described in this review. In this section, the safety and immunogenicity parameters involved in each AC category are discussed.

The result of CD4-PE40 clinical trials for HIV-1 cure exhibited unfavorable hepatotoxicity and the development of ADA with no apparent therapeutic benefits. Early speculation behind the observed hepatotoxicity of CD4-PE40 focused on the CD4 carrier moiety binding to free gp120 in the circulation leading to nonspecific uptake by hepatocyte (63), however, this was later refuted. Onda and colleagues found that the isoelectric point (pI) of the carrier molecule is positively associated with hepatotoxicity, and lowering the carrier molecule’s pI decreases the potential for hepatotoxicity (137). The high pI of CD4 (pI=8.86) could therefore explain the hepatotoxicity observed in animal studies and human trials. This was later addressed by the design of 3B3-PE38 with a carrier moiety exhibiting a much lower pI leading to no *in vivo* hepatotoxicity in rhesus macaques (118).

The issue of immunogenicity, especially for toxins, was later addressed by several strategies. This led to the truncation of the toxin (PE40 to a less immunogenic PE38), co-treatment with immune-modulating drugs, identification and manipulation of B and T cell epitopes, and other various recombinant modifications (138). Mutagenic deimmunization was also applied to DT, as well as deglycosylation of RAC to minimize interaction with hepatocytes (49, 139).

The development of ADA which could arise against the payload, linker, and the entire AC itself, is another major safety concern. The elicitation of ADAs could augment immune-related adverse events, anaphylactic reaction, and decreased potency of ACs. Ramachandran and colleagues observed the development of ADA against the carrier and payload for CD4-PE40, as well as the elicitation of antibodies neutralizing the construct (111). To track the development of ADAs, constant monitoring using highly sensitive assays are being used in cancer immunotherapy, which could then help inform the course of treatment, and therefore should be a crucial part of preclinical development for HIV-1 cure.

Antibody-radionuclide comes with a different set of safety considerations. The issue of the crossfire effect, notably to all constructs described herein, is a major concern. Current approved antibody-radionuclides, Zevalin and Bexxar, both FDA approved to treat non-Hodgkin’s lymphomas, contain beta-emitters and are both non-internalized. Beta particle-emitting radionuclides are characterized by the emission of high energy, sparsely ionizing, with ranges of up to a few millimeters. Due to this wider range of effects, beta emitters can induce a crossfire effect to neighboring, non-targeted cells. In contrast to a beta emitter, an alpha emitter radionuclide offers a much shorter range of radioactive decay, only traversing a few cells which are ideal for non-solid tumor-like targets. Alpha emitter radionuclide, as well as Auger radionuclide, both having shorter range, could therefore be an attractive choice to reducing crossfire effect. Furthermore, the *in vitro* characterization described in these studies observed cell crowding at the bottom of the cell culture plate, which could have potentiated the crossfire observed (45). Therefore, it is crucial to design new *in vitro* assays for crossfire determination.

The use of mAb subunits or nanobodies can also address concerns reading AC half-life. An intact IgG exhibits a longer circulation half-life which, depending on the conjugated payload, not be desirable (140). For radionuclide, for example, longer circulation half-life can promote undesirable radiation-absorbed doses to organs and blood which leads to myelotoxicity (141). Therefore, the availability of mAb subunits and nanobodies can be used to tailor a carrier of ACs depending on the intended circulation half-life requirements of a specific AC.

Antibody-oligonucleotide is a relatively new strategy and data on safety and immunogenicity is limited. The delivery of oligonucleotides has traditionally been the major limitation of its clinical applications. Strategies and modification of oligonucleotide for improved pharmacokinetics, pharmacodynamics, and biodistribution are an active area of research beyond the scope of this review (142). Two conjugation strategies were explored by AC for HIV-1, antibody-protamine fusion, and antibody-oligo-9-arginine peptide, both of which showed no toxicity in *in vitro* assay and *in vivo* mouse model (43, 143). An additional concern with oligonucleotide therapy is the possible off-target activation of intracellular TLRs, leading to an unwanted immune response. In mouse models, no TLR activation was observed, though careful monitoring in future studies is important (43, 69).

## Next-Generation AC for HIV-1

### Broadly Neutralizing Antibodies as Next-Generation Carrier

Most antibody carriers explored in the HIV-1 ACs employed murine, polyclonal, non-neutralizing, and/or first-generation neutralizing antibodies. However, the last decade saw increased innovation in the isolation and discovery of bNAbs with increased breadth and potency. BNAbs can be placed in a distinct category than ART because they directly target circulating virus, recognize Env-expressing infected cells, and can directly engage host antiviral responses such as ADCC. Several of these bNAbs have already entered various phases of human clinical trials for prevention, ART interruption, and therapeutic studies (144). Moreover, bNAbs are an attractive next-generation “armed” antibody for the delivery of molecular payloads for HIV-1 cure. The fundamental characteristics of bNAbs recognizing conserved epitopes across various clades and strains of HIV-1 remain of utmost importance providing selectivity as well as breadth, but additionally, the whole gamut of isolated bNAbs could offer increased flexibility, adaptability, and customization depending on the payload of choice.

The interest in bNAbs for clinical applications is indisputable, however, evidence reveals that not all bNAbs are created equal. Beyond their physical characteristics and binding specificities, bNAbs are diverse in terms of their function, and effectiveness in the context of therapeutic application. The requirement for neutralization of cell-free virions differs from those of cell-expressed viral proteins for effective ADCC. This can be explained by the various conformation of Env depending on the stage of infection, as well as the extensive viral heterogeneity that exists in the latent reservoir. The discovery and functional analysis of bNAbs for therapy is reviewed elsewhere (144, 145).

The functional variabilities of bNAbs make them an attractive carrier for ACs. As discussed throughout this review, the various mode of action of the payloads must be complemented by an appropriate mAb carrier moiety for optimal efficacy. Evidence provided that for toxins, carrier mAb targeting epitopes near the membrane is most effective (39, 136). However, these first-generation antibodies require sCD4 to be effective. In an indirect assay to screen bNAbs that make the best carriers of toxin, Pincus and colleagues found that bNAbs targeting CD4bs, V3 loops of gp120, and the gp41 HR/loop region to be effective as toxin carriers. Interestingly, not all gp41-targeting bNAbs showed efficacy as carriers with bNAbs targeting the MPER showed little to no efficacy as toxin carriers (59). This study therefore suggests that further characterization of bNAbs is required to elucidate the optimal carrier for ACs and that bNAbs targeting the CD4bs, V3 loops, and gp120-gp41 interface would be an interesting candidate for carriers of ACs.

For some ACs, rapid internalization is crucial for functionality. Therefore, antibodies that are readily internalized upon binding to their antigen are preferred. It has been reported that bNAbs targeting the Env “closed” conformation are readily internalized upon antigen-antibody ligation, in comparison to non-neutralizing anti-HIV-1 antibodies (146). The rapid internalization of Env in the closed conformation upon bNAb binding is a known evasion mechanism also seen in other viruses such as respiratory syncytial virus (RSV) and feline coronavirus infection (147, 148). This rapid antibody-Env internalization decreases the exposure time of bNAbs on the surface of infected cells, impairing host surveillance, and thus decreases the Fc-effector function of bNAbs. However, since ADCC is not the primary effector objective of ACs, this bNAb limitation for therapeutic use is not going to affect their efficacy as carrier molecules of ACs. Therefore, payloads such as toxins, oligonucleotide, and small drugs can be conjugated to bNAbs binding to the closed Env epitopes for rapid internalization. Meanwhile, non-neutralizing antibodies could be used for payloads with less dependence on internalization.

Several human trials have been conducted to test the safety and efficacy of bNAbs for therapy. In these clinical trials, bNAb infusion is deemed safe and well-tolerated without evidence of dose-limiting toxicity or adverse effect (149–152). Tested bNAbs were found to exhibit circulation half-life in the range of 9-13 days for HIV-1 infected individuals and 12-24 days for healthy subjects (144). The circulation half-life of bNAbs is an important factor for their efficacy as sufficient levels of bNAbs are needed to be maintained in the circulation to effectively neutralize and kill HIV-1infected cells. Moreover, certain mutations have been shown to further increase the half-life of bNAbs, without detrimental effect to its binding site and Fc-effector functions (153). BNAbs are also shown to have longer half-lives than receptor-targeting antibodies (144) which further makes them more advantageous than other carriers described herein. The safety and favorable pharmacokinetic profiles of bNAbs make them highly attractive carriers of ACs for HIV-1. This ensures increased circulation duration for ACs to reach their target sites. In case longer circulation half-life is not favored, bNAb subunits or nanobodies can be used.

### AC for HIV-1 Therapy

Evidence points to a multipronged approach for an effective cure for HIV-1. ACs could be a vital component in all stages of the HIV-1 treatment regimen. Although efficacies of bNAb monotherapy, bNAb in combination with ART and ART interruption studies, and bNAb in combination with LRAs are showing limited results in early human trials, the fact that these various combination treatments alongside bNAbs are all being tested for clinical efficacy could increase interest in ACs (144, 154, 155). In all these trials, bNAb infusion proved to be safe and well-tolerated which further highlights the advantage of mAb-based therapy over drug intensification approach for HIV-1 cure.

The modification of bNAbs as AC could further increase bNAb’s antiviral activity such as (1) neutralization of virus, (2) Fc-mediated effector function, (3) activation of endogenous host antiviral responses, with (4) directly delivering cytotoxic and immune-regulating payloads to infected cells.

ACs, like bNAbs, are likely to be transiently active limited by the pharmacokinetics and therapeutic window of their subunits. Therefore, ACs will require multiple infusions until all infected cells are purged. An active area of research focuses on a durable long-term expression of anti-HIV-1 antibodies *via* viral vector delivery or gene editing technologies (156). Additionally, the role ACs against cell-free virions remains to be established. In theory, during the acute phase of infection characterized by highly productive release of virions, the released virions can act as an antigen sink, decreasing potential AC-infected cell interaction (59).

Therapeutically, ACs could have the greatest efficacy for treatment during the acute infection phase of HIV-1 in complement with ART. After exposure, patients can undergo ART treatment immediately combined with ACs. This treatment strategy can simultaneously inhibit viral replication through the action of ART and eliminate infected cells through ACs. Clinicians can use information gathered from laboratory testing used to monitor ART responses such as plasma HIV-1 RNA and CD4+ T cell count to determine the appropriate carrier mAb and payload that may offer the best efficacy. In cancer immunotherapy, immunocompromised patients exhibited lower immunogenicity and adverse effect against antibody-toxins (157). If the same applies in the context of acute HIV-1 infection, it is worth exploring whether antibody-toxin is safe and effective during an active high-viremic HIV-1 infection in combination with ART. A lock-out approach *via* antibody-oligonucleotide could also be explored during acute infection by silencing host factors hijacked by HIV-1 such as the ESCRT machinery, coreceptors CCR5, and CXCR4, and HIV-1 genes preventing integration and viral maturation (158). Finally, a tandem antibody-based acute infection therapy could be explored by replacing ART entirely. As mentioned earlier, ACs role in neutralization of cell-free virion remains to be tested, however, unconjugated bNAbs in combination with conjugated bNAbs as ACs could potentially target both cell-free virions and infected cells, preventing the establishment and spread of viral reservoirs, respectively. This can be done by careful selection of bNAbs with high neutralization potency, and ACs with higher affinity to infected cells, both targeting non-overlapping epitopes on HIV-1 Env.

Since the involvement of cytotoxic effector cells are not primarily involved in the clearance of infected cells *via* ACs, during the chronic phase, LRA administration or ART interruption can be then followed by ACs. AC treatment can therefore act immediately after reactivation of latently infected cells, potentiating the “kill” in the shock-and-kill strategy. Antibody-TLR agonist conjugates would be an ideal construct during chronic HIV-1 infection as they can dually function as an LRA and engage Fc-effector function for the killing of reactivated cells. A lock-out AC construct with siRNA that can deliver epigenetic silencing can also be explored at during chronic infection (26). An example of such siRNA, developed by Suzuki and colleagues, targets the NF-κB sites in the HIV-1 promoter to induce potent transcriptional gene silencing (158). Lastly, ACs could be explored during virological blips observed in some patients. Blips are characterized by a sudden increase in viral RNA during suppressive ART. A recent study showed that endocervical endothelial cells release endogenous signals that can reactivate the latent reservoir (159). Therefore, passive administration of ACs during suppressive ART could potentially act immediately during virological blips, potentially decreasing the size of the viral reservoir.

To experimentally test the utility of ACs as a curative approach, preliminary *in vitro* cell line models of latency can be used. Cell lines such as U1 and ACH-2 are widely used HIV latency models and are suitable with reactivation by LRAs. However, these cell line models are limited in truly recapitulating the mechanism behind the actual HIV-1 reservoir in PLWH (160), therefore the best model would likely require *ex vivo* samples isolated from PLWH over established cell line models of infection and latency. Confounding differences in the Env conformation of various cell line models, integration site and method of establishing latency in latent cell lines, and the variability of Env expressed on released virions *versus* Env expressed on infected cell lines could obscure characterization results of ACs. Additionally, *ex vivo* isolated PBMCs can be isolated during acute phase of infection, upon ART treatment, ART naïve patients, and elite controllers to further elucidate the efficacy of ACs in various stages of infection. *Ex vivo* isolated PBMCs from PLWH can be further used to determine the optimal bNAb carrier that exhibits superior activity. The utility of ACs must overcome the safety and immunogenicity challenge of the first-generation ACs, and thus an appropriate safety, tolerability, and immunogenicity studies must be performed in a non-human primate model (NHP) model such as rhesus macaques. The established rhesus macaque persistence model can be adapted to determine the potency of ACs (161), either treated concurrently with ART, or during ART interruption or latency reversal.

## Discussion

As of the writing of this review, 14 new ACs are undergoing late phase clinical trials to test their efficacy against various cancer forms. These new ACs are employing the use of IgG carriers, as well as scFv, and bispecific antibodies. Additionally, these ACs are conjugating diverse payloads such as cytokines, toxins, inhibitors, and photosensitizers (antibodysociety.org). However, no HIV-1 ACs are being tested in human trials.

The high modularity of ACs for HIV-1 cure that was explored here is a double-edged sword. Although in theory, this makes ACs highly adaptable and customizable depending on the context of infection, this also makes them harder to characterize and investigate as a distinct therapeutic modality. This suggests that preclinical development, assay design, and validation will be different in each ACs.

The maximum potential of ACs for HIV-1 cure is currently untapped. Since the initial AC trials for HIV-1 three decades ago, the HIV landscape has significantly improved with dozens of approved ART, various preventative modalities, as well as increased diagnostics and surveillance. These advancement warrants a renewed interest in AC as a possible HIV-1 cure strategy that can address the limitations of ART. Furthermore, the HIV-1 AC field, though lacking relevant human trials experience, could piggyback in the thriving AC landscape in cancer immunotherapy, as well as the current enthusiasm for bNAbs in human trials. Therefore, it can be argued that an HIV-1 cure approach based on ACs will play a major role in the development of new HIV-1 cure therapies.

## Author Contributions

JU proposed the topic. JU created the tables and figures with input from ST. JU and ST structured the content, wrote, edited, and reviewed the manuscript. All authors contributed to the article and approved the submitted version.

## Funding

JU is supported by TKI-PPP grants Target2Cure by Health Holland/Aids Fund (LSHM19101-SGF) and Innovation Exchange Amsterdam (2019-1167). ST is supported by Young investigator grant P-53301 Aids fonds.

## Conflict of Interest

The authors declare that the research was conducted in the absence of any commercial or financial relationships that could be construed as a potential conflict of interest.
